# Chronic Kidney Disease Is Linked to Carotid Nodular Calcification, An Unstable Plaque Not Correlated to Inflammation

**DOI:** 10.14336/AD.2018.0117

**Published:** 2019-02-01

**Authors:** Marina Cardellini, Valentina Rovella, Manuel Scimeca, Lucia Anemona, Simone Bischetti, Sara Casella, Andrea Saggini, Elena Bonanno, Marta Ballanti, Francesca Davato, Rossella Menghini, Arnaldo Ippoliti, Giuseppe Santeusanio, Nicola Di Daniele, Massimo Federici, Alessandro Mauriello

**Affiliations:** ^1^Center for Atherosclerosis, Department of Medicine, Policlinico Tor Vergata, Rome, and Department of Systems Medicine, University of Rome Tor Vergata, Italy; ^2^Hypertension and Nephrology Unit, Department of Systems Medicine, University of Rome Tor Vergata, Italy; ^3^Department of Biomedicine and Prevention, University of Rome Tor Vergata, Italy; ^4^OrchideaLab S.r.l., via del Grecale 6, Morlupo, Roma, Italy; ^5^Department of Experimental Medicine and Surgery, University of Rome Tor Vergata, Italy; ^6^Vascular Surgery, Department of Biomedicine and Prevention, University of Rome Tor Vergata, Italy

**Keywords:** carotid, nodular calcification, chronic kidney disease, histopathology

## Abstract

The incidence and the different type of carotid calcifications, nodular and non-nodular, and their role in the acute cerebrovascular disease has not yet been defined. Various studies have correlated the presence of specific risk factors, in particular the chronic kidney disease, with the presence of calcification, but not with the type of calcification. Since it is likely that carotid nodular calcifications rather than those with non-nodular aspect may represent a plaque at high risk of rupture, the purpose of our study was to evaluate the role of nodular calcification in the pathogenesis of cerebrovascular syndromes and their possible correlation with specific risk factors. A total of 168 carotid plaques from symptomatic and asymptomatic patients submitted to endarterectomy, whom complete clinical and laboratory assessment of major cardiovascular risk factors was available, were studied. In 21 endarterectomies (5 from symptomatic and 16 from asymptomatic patients) an eruptive calcified nodule, consisting of calcified plates associated to a small amount of fibrous tissue without extracellular lipids and inflammatory cells, was found protruding into the lumen. Nodular calcifications were significantly observed in patients affected by chronic kidney disease (with GFR<60 ml / min / 1.73 m^2^), with a normal lipidic and glycemic profile. On the contrary, non-nodular calcification, mainly correlated to diabetes, were stable lesions. Results of our study suggest that the mechanisms and the clinical significance of carotid atherosclerotic calcification may be different. The nodular calcification could represent a type of unstable plaque, significantly related to chronic kidney disease, without inflammation, morphologically different from the classical vulnerable plaques.

Anatomic and clinical studies have demonstrated that acute cerebrovascular syndromes are pathogenically related to thrombosis and rupture of a carotid vulnerable atherosclerotic plaque, rather than only to the degree of stenosis [1-5]. Classically, a lesion classified as “vulnerable” identifies a plaque morphologically characterized by a large necrotic core with an overlying thin cap rich in inflammatory cells [6]. This "active" inflammation involves several types of blood cells, mainly T-lymphocytes, platelets and macrophages which are activated towards a pathway of inflammatory responses and secrete cytokines and lytic enzymes which, in turn, cause fibrous cap thinning, predisposing to plaque rupture [7-9]. The role of calcification in plaque rupture is still controversial [10-12].

Calcifications are a recognized prognostic factor for coronary artery disease [13-17] but still uncertain in cerebrovascular disease [18]. It is still not clear if calcification interferes with the stability of the carotid plaque or represents an age-related change without any clinical significance. Several studies evidenced an inverse correlation between the presence of calcium and the incidence of cerebral ischemic symptoms [18-19]. Other investigators stated that degree of plaque calcification may predict stroke risk, independent of stenosis grade [20].

In the modified AHA classification of coronary atherosclerotic plaques Virmani et al [6] have included in the “vulnerable plaques” the eruptive calcified nodules, a lesion with fibrous cap disruption associated with a non-occlusive thrombosis. This type of plaque represents a cause of thrombosis not correlated to cap inflammation. In this regard, an association between presence of calcified nodules and occurrence of thrombotic events have been demonstrated by the PROSPECT study, employing intravascular ultrasound analysis; nonetheless, results from this study revealed a lower number of major adverse events during 3 years of follow-up [21].

Usually, in stable plaques calcium shows a non-nodular aspect. It is likely that nodular calcifications rather than those with non-nodular aspect may represent a plaque vulnerability factor.

Calcified nodules were studied particularly in coronary circulation [6, 21-22]. Their incidence in the carotids and their role in the acute cerebrovascular disease has not yet been defined. Various studies have shown that some risk factors, such as hypertension, hypercholesterolemia and, particularly, chronic kidney disease (CKD) were significantly correlated with the presence of arterial calcification [23-25]. However, only few studies have investigated carotid district and did not distinguish nodular from non-nodular calcifications. The two types of calcification may also show a different elemental composition.

Therefore, the purpose of our study was to evaluate the role of nodular calcification in the carotid plaque destabilization and their possible correlation with specific risk factors.

## MATERIALS AND METHODS

### Cases selection

A total of 170 carotid plaques from symptomatic (major stroke or TIA) and asymptomatic patients submitted to surgical carotid endarterectomy (CEA) by patch reconstruction with use of shunt in case of EEG monitoring indication at the University of Tor Vergata (Rome, Italy), whom complete clinical and laboratory assessment of major cardiovascular risk factors was available, were studied. All asymptomatic patients showed a carotid stenosis >60%, assessed by echography or, in rare cases, by bilateral CT angiography.

Informed consent was obtained from each patient. The study protocol conforms to the ethical guidelines of the 1975 Declaration of Helsinki as reflected in a priori approval by the IRBs of our Institution.

### Histology

Only intact plaques were analyzed. Two carotid samples were excluded from the study because of fragmentation damage. Therefore, 168 plaques were included in the study and histologically analyzed. The sampling collection and analysis methods have been previously reported [1]. Briefly, samples were fixed immediately upon removal in 10% buffered formalin for 24 hrs. All plaques were cut transversely every 5 mm, embedded in paraffin and stained with haematoxylin-eosin. The paraffin blocks with a greater shear strength underwent to a surface decalcification process. Normally, the paraffin block placed face down in a decalcifier (LEICA Surgipath Decalcifer II) for 15 - 60 minutes after trimming and when the calcium is discovered. This process allowed to decalcifier acid to penetrate just few layers into the block to dissolve the calcium. After treatment, the paraffin block was thoroughly rinsed in water to remove residual acid, chilled and sectioned. Finally, a careful realignment was required because the decalcifier acid has a low penetrance allowing to cut only a couple of sections.

### Plaque classification

Plaque were divided, according to the presence and type of calcification, in to two groups: (a) those with nodular calcification and (b) those without nodular calcification.

Plaque with nodular calcification were defined those characterized by eruptive calcified nodules protruding into the vascular lumen associated to a very small amount of fibrous tissue without extracellular lipids or a necrotic core.

Plaques without nodular calcification were classified, according to the modified American Heart Association atherosclerosis classification [6], into stable and unstable. Unstable plaques included (a) thrombotic plaques associated with rupture or erosion of the cap; (b) healed plaque with a thrombus in organization; (c) vulnerable plaque or thin-cap fibro-atheroma (TCFA) characterized by a fibrous cap less than 165 µm thick heavily infiltrated by macrophages, CD68 positive (>25 per high magnification field), without plaque rupture [26]. Stable plaques were divided in fibroatheroma and fibrocalcific. Fibroatheroma consists of an acellular necrotic core covered by a thick fibrous cap (> 165 µm) consisting mainly of smooth muscle cells. Fibrocalcific plaques were mainly constituted by fibrous tissue associated to a variable necrotic core and a large not-eruptive calcification.

Plaques with nodular calcifications were not divided into stable and unstable plaques because one of the purposes of this study was to verify whether such plaques should be regarded as unstable plaques as a whole, irrespective of other features.

Histopathologic examination was performed by two different pathologists (AM, LA) blinded to the clinical data utilizing the definitions reported below. Interobserver reliability was >98%.

### EDX microanalysis

One cm in diameter and 1 cm in length of 5 plaques with macroscopic evidence of an eruptive calcification and 5 fibrocalcific plaques were fixed in in 4% (v/v) paraformaldehyde for 24h. The samples were treated as previously described [27]. Critical point drying (Agar Scientific, Essex, UK, Elektron Technology UK Ltd., Cambridge, UK) with supercritical CO_2_ was then performed to prevent cell deformation. EDX microanalysis was performed by using a liquid N_2_-cooled Si detector with a super-ultrathin Be window on unpost-fixed samples placed on specific copper stubs. Spectra were collected by a SEM LEO 1450VP (Carl Zeiss Meditec, Oberkochen, Germany) scanning electron microscope at acceleration voltage of 5 KeV employing an area scan mode (640 × 640 μm sampling area), 300 s acquisition time, and 32-37% detector dead time. Analysis of acquired spectra was performed under a nonstandard mode using atomic number-absorption-florescence correction (ZAF) methods using Inca Energy software (Oxford Instruments Ltd., High Wycombe, UK; Si(Li) detector, ATW - atmospheric thin window, resolution 133 eV for MnKα at 10 000 counts). For each specimen, we acquire 5 spectra on 8 mm^2^ of calcification surface in total.

### Risk factors definition

Clinical records were reviewed for all cases in order to determine risk factor profile. The risk factors were defined utilizing the following criteria: (a) hypertension: patients with positive clinical history of systolic BP > 140 mmHg and/or a diastolic BP > 90 mmHg without antihypertensive treatment or taking antihypertensive treatment at the time of carotid endarterectomy; (b) diabetes mellitus: patients with fasting blood glucose > 126 mg/dL and/or following oral treatment or insulin therapy; (c) patients with tobacco dependence were categorized as smokers and former smokers. Former smokers who had stopped smoking for less than five years were considered as smokers and patients who had not smoked for >5 years were considered as non-smokers; (d) hypercholesterolemia: patients with total cholesterol level > 200 mg/dL (> 5.18 mmol/L); (e) patients with low HDL-C: < 40 mg/dL in men or < 50 mg/dL in women [28]; (f) hypertriglyceridemia: patients with serum triglycerides levels > 150 mg/dL (> 1.70 mmol/L ) [28]; (g) abdominal obesity (patients with a waist circumference ≥102 cm in men or ≥88 cm in women.

In order to evaluate levels of atherogenic cholesterol [29-30], low-density lipoprotein cholesterol (LDL-C) was calculated by the Friedewald equation [31]: LDL-C = cholesterol - (HDL-C + (triglycerides/5)). A value of LDL-C of> 100 mg/dL was utilized has cut-off between high and low levels.

In addition, estimated glomerular filtration rate (eGFR) was defined and calculated using the CKD-EPI (chronic kidney disease epidemiology collaboration) equation. Values of eGFR<60 mL/min per 1.73 m^2^, present for >3 months, were considered as cut-off for renal chronic disease (GFR categories: G3a-G5) [32].

### Statistical analysis

Data were analyzed using SPSS version 16.0 (SPSS Inc, Chicago, Ill) software. Continuous variables were expressed as the mean ± SD. The Shapiro-Wilk test was used to statistically assess the normal distribution of the data. Comparisons between continuous variables were performed using the independent Student t-test or the Wilcoxon rank sum test. Categorical data were analysed using the chi square test or the Fisher exact test.

Multivariate analysis using stepwise logistic regression (using the “enter” method for variable selection, adjusted for age, sex) was utilized to identify independent risk factors which significantly correlate with the presence of nodular calcification. The following variables were included: age, gender, hypertension, diabetes, smoking habit, high LDL-C, obesity and low eGFR. Multivariate analysis was performed in 2 models: (1) nodular calcification vs. stable and fibrocalcific plaques; (2) nodular calfications vs. unstable plaques.

A 2-tailed p value <0.05 was considered statistically significant.

**Table 1:** Baseline characteristics of patients.

	*N (%) or mean (SD)*
Total	N = 168
Age	73.05 (8.47)
Gender	
Male	123 (73.2%)
Female	45 (26.8%)
Cerebrovascular disease	
Symptomatic patients	42 (25.0%)
Ipsilateral major stroke	21 (12.5%)
TIA	21 (12.5%)
Asymptomatic patients	126 (75.0%)
Risk factors	
Hypertension	145 (86.3%)
Diabetes	71 (42.3%)
Smoking habit	82 (48.8%)
Low-density lipoprotein cholesterol (LDL-C) (>100 mg/dL)	65 (38.7%)
Low eGFR (<60mL/min per 1.73 m2)	53 (31.5%)
Obesity (BMI >30 kg/m2)	26 (15.5%)
Drugs	
Statins	100 (59.5)
Diuretics	66 (39.3)
Associated vascular disease	
Previous myocardial infarction	34 (20.2)
Peripheral arterial disease	53 (31.5)
Aortic aneurysm	10 (6.0)
Histological type of carotid plaque	
Plaques with nodular calcification	21 (12.5%)
Plaques with non-nodular calcification	147 (87.5%)
Stable plaques	76 (45.2%)
Fibroatheroma	25 (14.9%)
Fibrocalcific	51 (30.3)
Unstable plaques	71 (42.3%)
Thrombotic plaque	31 (18.5%)
TCFA	12 (7.1%)
With a thrombus in organization	28 (16.7%)

## RESULTS

### Clinical data

The clinical characteristics of patients are reported in the Table 1. The mean age of 168 patients at time of surgical carotid endarterectomy (CEA) was 73.05 + 8.47 years. Of those 123 (73.2%) were male, and 45 (26.8%) female; 42 patients (25.0%) were symptomatic (affected by ipsilateral major stroke or transient ischemic attack), while 126 (75.0%) were asymptomatics who underwent CEA for high grade carotid stenosis.

All patients included in the study had at least one risk factor. The hypertension was the risk factor most frequently observed (in 145 patients, 88.9%). In particular, 61 patients (35.7%) showed high eGFR (Table 1). Continuous treatment with aspirin (100 md/die) was administered to all patients in the post-operative and follow-up periods.

### Histopathological findings

**Figure 1.**
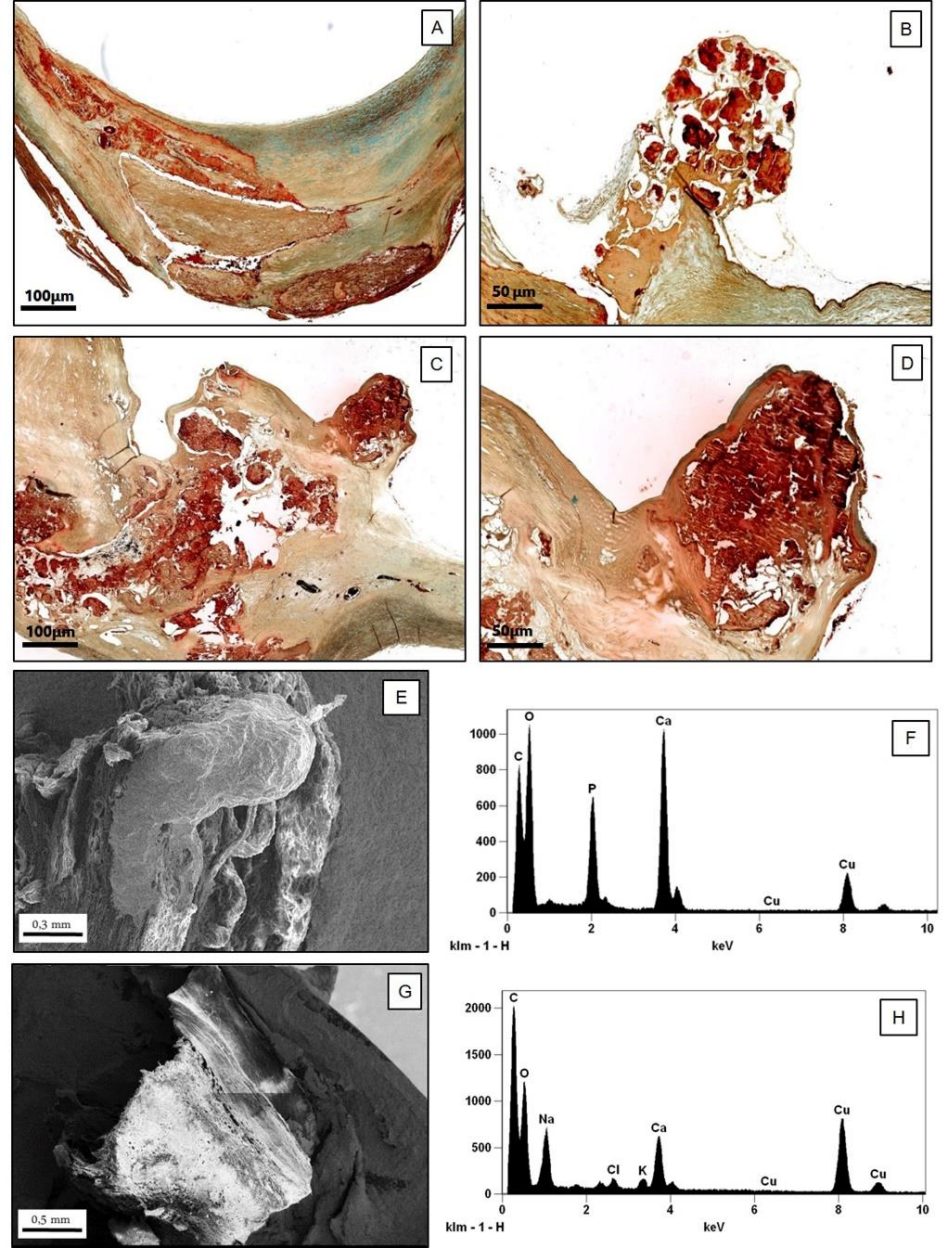
**Characterization of nodular calcification. A-D) Histological and ultrastructural characterization of calcifications in carotid plaques**. (A) Stable fibrocalcific plaque with a linear calcification (Movat staining,2x); (B) Calcific carotid nodule protruding into the lumen consisting of calcified plates associated to a small amount of fibrous tissue without extracellular lipids, necrotic core and inflammatory cells. The fibrous cap over the nodule was extremely thin (Movat staining, 5x); (C, D) Images display a calcific nodule associated to fibrin deposition with a small superficial ulceration (C): Movat staining, 2x, D (particular of C): 6x). (E-H) Scanning electron microscopy and EDX microanalysis of carotid plaque: (E) The image shows an eruptive calcific nodule with disruption of fibrotic cap (scanning electron microscopy, 100x); (F) EDX spectrum shows that this calcific nodule is made from hydroxyapatite; (G) Linear calcification in a stable fibrocalcific carotid plaque (scanning electron microscopy, 90x); (H) EDX spectrum demonstrates that this linear calcification is made from calcium oxalate.

In 21 CEA (5 from symptomatic and 16 from asymptomatic patients) a nodular calcified nodule was found protruding into the lumen (Table 1, Fig. 1B-D). In none of these cases inflammatory cells were found (Fig. 1B). The fibrous cap over the nodule was extremely thin (Fig. 1B) and sometimes fibrin deposition within the nodule was identified (Fig. 1C, D). In 5 of 21 cases, a discontinuity of the fibrous cap with loss of endothelial cells was observed with an overlying non-occlusive luminal thrombus identified (Fig. 1C, D); importantly, all such 5 cases belonged to the symptomatic group of patients. In the remaining 16 cases a complete endothelial cell coverage over the nodule was clearly shown. Thirteen calcified nodules were observed at the level of the carotid bifurcation, 2 in the common carotid artery and 6 in the internal one. They had always an eccentric localization and only 6 cases determined a luminal stenosis> 70%. In these 6 cases they were located in the segment with the most severe stenosis, while in the remaining 10 they were found either before or after the narrowest stenosis.

In 71 patients (36 symptomatic and 35 asymptomatic) unstable plaques rich in inflammatory cells were found, consisting in 31 thrombotic plaques (all from symptomatic patients) associated with rupture of a thin and inflamed fibrous cap, 12 thin-cap fibro-atheroma TCFA (6 from symptomatic and 6 from asymptomatic patients) and 28 plaques with an organizing acute thrombus (5 from symptomatic and 23 from asymptomatic patients) with the typical network of large, thin-walled vascular channels and a variable number of macrophagic cells loaded with hemosiderin within the area of an acute thrombus (Table 1).

**Table 2:** Nodular calcifications vs. stable fibrocalcific plaques.

	Nodular calcification N= 21	Stable fibrocalcific plaques N = 51	Uni variate Analysis P	Multi variate Analysis P
Age, *mean (SD)*	74.6 (11.3)	74.2 (7.2)	0.90	0.89
Gender M F	15 (71.4%) 6 (28.6%)	36 (70.6%) 15 (29.4%)	0.61	0.94
Hypertension	17 (81.0%)	48 (94.1%)	0.09	0.21
Diabetes	6 (28.6%)	28 (54.9%)	0.04	0.03*
Smoking habit	9 (42.9%)	25 (49.0%)	0.63	0.37
High LDL-C *(>100 mg/dL)*	3 (14.3%)	16 (31.4%)	0.13	0.21
Low eGFR *(<60 mL/min per 1.73 m^2^)*	12 (57.1%)	16 (31.4%)	0.04	0.03
Obesity *(BMI >30 kg/m^2^)*	1 (4.8%)	12 (23.5%)	0.06	0.11

* In the logistic regression value of B showed the minus sign

The remaining 76 carotids (45.2% of cases) showed a stable plaque. Fifty-one of these were fibrocalcific and had a thick, fibrous cap overlying extensive accumulations of calcium in the intima close to the media, associated to a small lipid-laden necrotic core. In this type of plaque, unlike the calcified nodules, calcium had a non-nodular aspect (Fig. 1A) and not protruded inside the lumen as an eccentric nodular relief. The other stable plaques were constituted by 25 fibrous cap atheromata, characterized by a large lipid-necrotic core containing extracellular lipid, cholesterol crystals and necrotic debris covered by a thick fibrous cap with few inflammatory cells (Table 1).

### Nodular calcifications vs. stable fibrocalcific plaques

Univariate analysis demonstrated that patients with a carotid nodular calcification, compared to those with stable fibrocalcific plaques, showed a significant reduction of the eGFR (p=0.04). In fact, 57.1% (12/21) of patients with nodular calcification was suffering from CKD, compared with 31.4% (16/51) of the patients with stable plaques (Table 2). It is noteworthy that only 3 patients with calcified nodules (4.8% of cases) had high values of LDL-C and only one was obese (with BMI>30 kg/m^2^).

Multivariate logistic regression confirmed that a lower eGFR was the most important independent factor associated to the presence of calcified nodule (p=0.03). On the contrary, the presence of diabetes that was significantly observed in patients with stable plaques (54.9% vs. 28.6%, p=0.03) (Table 2).

When the subgroup of patients with calcify nodules was compared with the whole subgroup of patients with stable plaques (fibrocalcific and fibroateroma), the results obtained by multivariate analysis further demonstrated that the CKD was significantly correlated with the presence of calcific nodules (p=0.02), while the diabetes with that of stable plaques (p=0.02).

### Nodular calcifications vs. unstable plaques

When the plaques with nodular calcification were compared with those unstable (plaque with an acute or with a thrombus in organization; TCFA), univariate analysis showed that patients with unstable plaques had significantly higher levels of LDL-C (p=0.005). Multivariate analysis confirmed this result since the most significant independent factor that correlated with unstable plaques, as compared to nodular calcifications, was the presence of high values of LDL-C (p=0-01) (Table 3).

**Table 3:** Correlation between nodular calcification and unstable plaques.

	Nodular calcification N= 21	Unstable inflamed plaques N = 71	Uni-variate Analysis P	Multi- variate Analysis P
Age, *mean (SD)*	74.6 (11.3)	71.3 (8.5)	0.16	0.47
Gender M F	15 (71.4%) 6 (28.6%)	59 (83.1%) 12 (16.9%)	0.35	0.04
Hypertension	19 (90.5%)	61 (85.9%)	0.73	0.83
Diabetes	6 (28.6%)	26 (36.6%)	0.61	0.20
Smoking habit	9 (42.9%)	38 (53.5%)	0.46	0.41
High LDL-C *(>100 mg/dL)*	3 (14.3%)	36 (58.1%)	0.005	0.01
Low eGFR *(<60 mL/min per 1.73 m^2^)*	12 (57.1%)	18 (25.4%)	0.009	0.12
Obesity *(BMI >30 kg/m^2^)*	1 (4.8%)	10 (14.1%)	0.45	0.23

### EDX microanalysis

Area with calcified nodules were identified in blue toluidine semi-fine sections. SEM analysis performed on selected area showed two morphological patterns of calcification. In all samples we observed large crystal calcium deposits with 1 mm diameter. EDX analysis allowed us to find in nodular calcification that both micro and macro calcifications were made of hydroxyapatite (HA) (Fig. 1E, F). In particular, the EDX detector captured X-ray Kα 3.91 KeV and Le 0.341 for calcium (Ca), X-ray Kα 2,013 KeV for phosphate (P) and X-ray Kα 1,041 KeV for sodium (Na). The *ratio* between Ca and P X-ray count were comparable with HA standard sample. On the contrary, calcium-oxalate was mainly reported in fibrocalcific plaques (4 out of 5 cases). From ultrastructural point of view, oxalate calcifications appeared associated to linear calcifications (Fig. 1G, H).

## DISCUSSION

Results of our study have shown that nodular calcifications were significantly observed in patients affected by CKD, documented by a value of eGFR <60 ml/ min/1.73 m^2^ (GFR categories: G3a-G5).

The objective of our study was to evaluate the role of calcified nodules in the plaque destabilization, rather than the simple correlation with the cerebrovascular events, as the target for the prevention of ischemic cerebrovascular events is represented by the identification of the plaque “vulnerable to the rupture”, before this gives rise to clinical symptoms. We believe that our findings may be of great help to identify patients at risk prior to the acute event.

Many previous studies shown the link between CKD and artery calcification, in particular concerning coronary artery calcification (CAC) [13-17]. In this study we also demonstrated a significant correlation with the calcification of the carotid district, which had previously been evaluated mainly by imaging [23,25]. Specifically, for the first time, we have shown a correlation with the presence of carotid calcified nodules that represent a type of calcification at high-risk for plaque rupture.

Our data appear to suggest that nodular calcification, despite not being a frequent lesion in the carotid district, might be the hallmark of an unstable plaque subtype, devoid of inflammation, morphologically different from conventional vulnerable plaques. In all cases, nodular calcification consisted of an eruptive, dense, calcified mass protruding into the lumen with an irregular surface; lesions were eccentric in most cases (Fig. 1B-D). The observation that 5 out of 21 cases showed a discontinuity of the thin fibrous cap associated with an overlying luminal thrombus seems to corroborate the hypothesis that plaques with nodular calcification should be regarded as unstable. These lesions correspond to the “calcified nodule” reported by Virmani et al. [6]. The remaining 16 plaques showed a similar morphological appearance despite lack of association with thrombosis. As no significant differences were observed between plaques with nodular calcification with or without an overlying acute thrombus as regards location within the carotid district, vascular stenosis, and histological appearance, we believe that any plaques featuring nodular calcification, even in absence of an overlying acute thrombus, ought to be regarded as unstable plaques, at high risk of rupture and thrombosis.

Results of our study hypothesize the presence in the carotid district of two different types of unstable plaques, the first represented by the "classical" TCFA in which inflammation is the pathogenic mechanism that determines the rupture of the cap, the latter constituted by nodular calcifications unrelated to inflammation. While TCFA and other unstable inflamed plaques are significantly related to the presence of an altered lipidic profile, as stated by significantly higher levels of LDL-C (Table 3), the calcified nodules were observed in patients with normal lipidic profile and with chronic renal failure.

The presence of calcifications is very common in aged atherosclerotic lesions. Although the mechanisms of calcification remain poorly understood, the presence of apoptotic cells, extracellular matrix, and necrotic core material may promote the deposition of microcalcifications, which can subsequently form extensive calcium deposits in the plaque [14, 33-34]. The necrotic core often can completely calcify. In this type of plaques, classified as fibrocalcific, the calcification, unlike the nodular calcifications, shows a laminar pattern, does not protrude inside the lumen and not cause rupture of the cap (Fig. 1A). Apart from the methods used to quantify calcium, various histological studies have confirmed that the fibrocalcific plaque gives stability to the carotid artery and is associated to absence of neurologic symptoms [18-19]. Vulnerable plaque tends to be either not calcified or with only mild to moderate calcification, suggesting that calcification may exert a protective effect [35]. The nodular calcifications, unlike the fibrocalcific plaques, usually protrude into the lumen and could induce flow disturbances, causing a modification of the laminar blood flow into a disturbed or oscillatory flow with an irregular distribution of wall shear stress, making the plaque highly susceptible to rupture [36-37].

Chronic kidney disease represents the most important risk factor associated to the nodular calcification. Vascular calcification is very frequent in patients with CKD and represents an independent predictive factor of future cardiovascular events and mortality [38]. Vascular calcification is highly prevalent in patients with end-stage renal disease and has an important clinical significance as the cardiovascular morbidity and mortality among dialysis patients are substantially higher than in the general population [39-40]. The CAC is significantly higher in dialysis patients than in age- and gender-match subjects from the general population [41]. Moreover, patients with progressive CKD show an early development of CAC, as reported by Goodman et al. [41] which demonstrated that an early CAC is common and progressive in young adults with end-stage renal disease who are undergoing dialysis. Nevertheless, the CAC develops early also in non-dialyzed patients with CKD [42].

The medial calcification is considered the more common and major form of calcification in patients with CKD [43] and appears to be constituted by thick hydroxyapatite crystals [44]. To this end, two different types of calcific vasculopathy, inflammatory versus metabolic, have been hypothesized by Demer and Tintut [45], the first associated with calcification of necrotic lipid core that occurs in the aged atherosclerotic plaques and the latter correlated with the CKD and calcification of the tunica media.

Result of our EDX microanalysis demonstrates that the composition of intimal nodular calcification is similar to that of medial calcification, both being constituted by hydroxyapatite (Fig. 1E-F). On the contrary, calcification found in the fibrocalcific plaque are mainly constituted by calcium-oxalate (Fig. 1G-H). As concern elemental composition of calcification, studies reported two different forms of calcium salts, calcium oxalate and hydroxyapatite [46-47]. Generally, the calcium oxalate deposition was associated to degenerative process [48] whereas various studies showed how hydroxyapatite calcification was a highly regulated and organized active cellular process [49]. The precise mechanism responsible for vascular calcification in CKD and the contribution of impaired bone metabolism to vascular calcification have not been fully elucidated. This type of calcification is independent of inflammation and results from a modification of vascular smooth muscle cell phenotype. A key to vascular calcification is the differentiation of contractile vascular smooth muscle cells into a chondrocyte or osteoblast-like cells and is regulated by active inducers and inhibitors. Inhibitors of vascular calcification, such as matrix gla protein, osteopontin and fetuin-A are down-regulated in CKD [34, 45]. Moreover, in arteries from CKD patients the expression of the Runt-related transcription factor 2 (Runx2), the key transcription factor that regulates osteoblast differentiation and chondrocyte maturation was identified suggesting an important role of Runx2 in the pathogenesis of vascular calcification associated to CKD [50]. Overexpression of TNFα, could activate the osteochondrogenic program in vascular smooth muscle cell [51]. In our cases patients with eGFR showed a significantly increase of TNFα as compared to those with normal eGFR (17.61 + 2.5 vs. 12.10 +1.74, p= 0.03).

Patients with medial calcification had less conventional risk factors for atherosclerosis [39, 43]. Similarly, in our cases nodular calcification occurs in patients with normal lipidemia and normal glycemic profile. In fact, only 3 out of 21 (14.3%) patients with nodular calcification showed high LDL-C and only 6 (28.6%) were diabetics.

Our results hypothesize a different pathogenic mechanism for different types of plaque calcification. It can be hypothesized that inflammation may play a role in the non-nodular plaque calcification. Recent studies provided numerous evidences that vascular calcification is correlated with inflammatory status and is enhanced by inflammatory cytokines [45, 52-54]. In a preclinical model Aikawa et al [52] demonstrated in vivo by molecular imaging that osteogenesis is associates with inflammation in early-stage atherosclerosis. In addition, in a longitudinal FDG-PET/CT study authors demonstrates a close relationship between chronic inflammation and vascular calcification since focal arterial inflammation precedes subsequent calcification in the same location [45, 52-54]. Inflammation could also mediated the significant correlation that we found between the presence of a non-nodular calcification and that of diabetes, as reported in Table 2. Vascular calcifications are very frequent in subjects with diabetes mellitus [14, 34, 45]. Several mechanisms may be involved. One is the formation of advanced glycation end products (RAGE) and it has been demonstrated that the receptor for RAGE colocalizes with microcalcifications and inflammatory cells in the plaque [45, 55].

### Study limitation

The major limitation of this study is that we examined only carotid plaques from patients who underwent carotid endarterectomy. However, this is an intrinsic limitation of the histological methodology. Since this study was performed on CEA, an obvious limitation is the lack of follow-up data on these patients which would have helped in proving or disproving the hypothesis that carotid nodular calcification represents an unstable plaque.

### Conclusions

Results of our study suggest that the mechanisms and the clinical significance of carotid atherosclerotic calcification may be different. Nodular calcification, correlated to presence of CKD, probably are high-risk lesions. On the contrary, the majority of plaques with non-nodular calcification are low-risk lesions. Considering the fact that a remarkable number of asymptomatic patients or patients who have non-specific symptoms are submitted to prophylactic CEA, the identification of risk profile and morphological characteristics of plaque calcification by non-invasive methods will help to identify either patients at higher stroke risk or those with the most favourable outcome profile.
